# Retrospective analyses of dogs found serologically positive for *Ehrlichia canis* in Cebu, Philippines from 2003 to 2014

**DOI:** 10.14202/vetworld.2016.43-47

**Published:** 2016-01-13

**Authors:** Adrian P. Ybañez, Rochelle Haidee D. Ybañez, Rex R. Villavelez, Honey Pearl F. Malingin, Dana Natasha M. Barrameda, Sharmaine V. Naquila, Shiella Mae B. Olimpos

**Affiliations:** 1 Biology and Environmental Studies Program, Sciences Cluster, University of the Philippines Cebu, Lahug, Cebu City, Philippines; 2 Southwestern University, Villa Aznar, Urgello St., Cebu City 6000, Philippines; 3 GPY Veterinare Animale – Group of Veterinary Clinics, Cebu City 6000, Philippines

**Keywords:** Cebu, clinical signs, dog, *Ehrlichia canis*, hematology, Philippines

## Abstract

**Aim:**

The study aimed to document the clinical and hematological observations of dogs found serologically positive for *Ehrlichia canis* and to identify parameters or factors that are associated with the disease with focus on the anemic and thrombocytopenic state of the infected dogs.

**Materials and Methods:**

From 7 participating veterinary establishments, a total of 913 cases from 2003 to 2014 were initially assessed using inclusion criteria, including *E. canis* diagnosis by the attending veterinarian and the presence of ticks or history of infestation, thrombocytopenia, and/or anemia. From these, 438 cases that were found serologically positive for *E. canis* using commercial test kits were selected. Profile, clinical observations and hematological test results were obtained from the selected cases. Computations for statistical associations between the anemic and thrombocytopenic state of the infected dogs and their profile, observed clinical signs and other hematological values were performed.

**Results:**

Most of the dogs were purebred (60.0%) and female (51.1%) and were within the age range of 1-5 years (38.4%). The mean packed cell volume (PCV), red blood cell (RBC) count, and platelet count were lower than the normal values while the absolute count of basophils were higher than normal values. Creatinine and blood urea nitrogen (BUN) appear to be elevated. The most common clinical signs observed were inappetence (41.3%), lethargy/depression (35.6%), vomiting (32.4%), fever (18.5%), paleness (8.2%), and epistaxis (6.6%). Analyses showed that there were no significant differences on the hematological values and clinical signs between thrombocytopenic and non-thrombocytopenic seropositive dogs. Moreover, very weak correlations between platelet count and RBC count, absolute lymphocyte count, and neutrophil count were found. On the other hand, only paleness (p=0.008) and epistaxis (p=0.004) were found to be significantly different between anemic and non-anemic patients. This coincided with the linear regression results where PCV (p=0.000, R=0.787, R^2^=0.619) was moderately correlated with the RBC count. In addition, eosinophil count was found weakly correlated.

**Conclusion:**

*E. canis* infection in dogs may produce varied clinical signs that may be influenced by the thrombocytopenic and anemic states of affected animals. Complete blood counts remain important in the diagnosis of the disease, especially the platelet and RBC counts. Creatinine, BUN and alanine aminotransferase can be of value in the diagnosis of the infection. Several cases were lost to follow-up and appeared to be a challenge for handling veterinarians to monitor compliance of owners and progress of infected patients.

## Introduction

Canine ehrlichiosis or canine monocytic ehrlichiosis (CME) is a disease of major global significance that affects dogs. It is caused by *Ehrlichia canis*, a Gram-negative, coccoid to ellipsoidal, often pleomorphic, intracytoplasmic bacteria belonging to the family Anaplasmataceae [1]. It is transmitted by the ubiquitous brown dog tick, *Rhipicephalus sanguineus* complex [2,3]. Recently, *E. canis* has been detected in humans [4]. It infects the monocytes, granulocytes, and platelets [5].

*E. canis* may be detected together with *Anaplasma platys*, a bacteria transmitted by the same tick vector. Co-infection with this pathogen can lead to severe clinical signs [2,6]. CME can also produce non-specific clinical signs [5]. As CME can be a multi-systemic disease [6], it may manifest several clinical signs that can lead to disease misdiagnosis.

Molecular and serological evidence have validated the presence of *E. canis* in the Philippines [5,7-10]. As it can be life threatening, its timely recognition to institute immediate and appropriate therapy is vital for the survival of the affected animal [5]. Except for a case report by Ybañez [5], there are no other published researches documenting the clinical signs of CME cases in the Philippines. Collecting and gathering information detailing the most common clinical signs manifested in the area can be beneficial. Hence, this study endeavored to determine the profile of dogs and clinical signs that may be correlated with the thrombocytopenic and anemic states of infected dogs and assess the applicability of the neutrophil to lymphocyte ratio in predicting the outcome of CME disease.

## Materials and Methods

### Ethical approval

The study was performed in accordance with the Institutional Animal Care and Use Committee guidelines of Southwesern University, Cebu and the University of the Philippines Cebu, and with the approval of the attending veterinarians, chief veterinarians and/or proprietor of the veterinary establishments. The study relied on records and no animal was used in the conduct of the study.

### Veterinary establishments

Several animal clinics and hospitals in Cebu, Philippines were invited to participate, but only 7 positively responded: (1) GPY Veterinare Animale-Main Branch, Cebu City, (2) AZYP Pet Doctor’s Veterinary Center, Talisay City, (3) Pet Science Veterinary Center, Cebu City, (4) Animal Kingdom Veterinary Hospital, Cebu City, (5) George Animal Clinic, Lapu-Lapu City, and (6) Mactan Animal Clinic, Lapu-Lapu City, Cebu, Philippines.

### Selection of CME cases

A total of 913 cases from 2003 to 2014 were reviewed using inclusion criteria, including *E. canis* diagnosis by the attending veterinarian and the presence of ticks or history of infestation, thrombocytopenia, and/or anemia. From these, 475 and 438 cases were, respectively, considered as “suspected” and “validated” CME cases depending on whether or not the animal patients were tested for *E. canis* using a commercial test kit (Immunocomb^®^, Biogal, Israel), with a reported sensitivity and specificity of 86% and 98%, respectively [6]. Using a fixed survey form, the profile, clinical history, and laboratory test results of the validated CME cases were obtained. Moreover, presenting clinical signs, which were dependent on the attending veterinarian’s observations, were also obtained from the clinical records.

### Data processing and analysis

Data from the fixed survey form were manually tabulated in a tally sheet and then encoded to Microsoft Excel using appropriate coding to facilitate statistical analyses. Common clinical signs were identified while laboratory test results were summarized. Cases were grouped based on their anemic and thrombocytopenic states and were tested for associations against profile, laboratory test results and presenting clinical signs using Chi-square, logistic regression, analysis of variance and general linear model multivariate analyses.

## Results and Discussion

Most of the dogs were of pure breed (60.0%), female (51.1%), and within the age range of 1-5 years (38.4%) (Table-1). In a study by Akhtardanesh *et al*. [11], no breed and sex predilection were found in seropositive dogs. Similarly, Harrus *et al*. [12] observed no age predilection in CME cases. In contrast to other studies, certain breeds were identified to be predisposed to certain clinical signs [13], like the case of German shepherds showing hemorrhagic signs (including epistaxis), and the Beagles and mongrels showing typical signs of the disease [14]. Further studies are needed to determine breed predispositions in the Philippine setting. On tick infestation, only 19.9% of the patients were noted to have the parasites, which is lower than previously reported (52.7%) [15]. This indicates that the dog patients may have been exposed to ticks and the pathogen earlier. Thus, in CME diagnosis, the absence of ticks in the observed patient cannot rule out the possibility of infection.

**Table-1:** Profile of *E. canis*-seropositive dogs in Metro Cebu, Philippines (n=438).

Parameter	N (%)
Breed	
Pure	263 (60.0)
Mixed	143 (32.6)
None-specified	32 (7.3)
Sex	
Female	224 (51.1)
Male	183 (41.8)
Not specified	31 (7.1)
Age	
>1	70 (16.0)
1-5 years	168 (38.4)
Above 5 years	59 (13.5)
Not specified	141 (32.2)
Mean: 3.6 years, SD=10.9	
Tick infestation	
Noted	87 (19.9)
Not noted	351 (80.1)
Recorded outcome	
Recovered	133 (30.4)
Dead	29 (6.6)
Lost to follow-up	276 (63.0)
Thrombocytopenic	
Yes	316 (72.1)
No	45 (10.3)
Lost data	77 (17.6)
Anemic	
Yes	234 (53.4)
No	115 (26.3)
Lost data	89 (20.3)

*E. canis=Ehrlichia canis*, SD=Standard deviation

Only 33% were noted to recover from the disease after treatment. In subclinical infections, dogs are able to spontaneously recover from the disease without treatment [16]. As more than the majority of the patients were lost to follow-up (63.0%), it remains a big challenge for veterinarians to ensure that owners follow the suggested treatment protocol and allow pets to recover from the disease. If treatment is discontinued, infected dogs may enter into the chronic phase of CME. In chronic cases leading to death, treatment appears to have no effect to the outcome of the disease [16].

A high proportion of the dogs were thrombocytopenic and anemic (at least 72.14% and 53.4%, respectively). Thrombocytopenia and anemia are considered the hallmarks of CME infection. Pancytopenia and leucopenia (only 5.7% and 7.3%, respectively, in this study) reported by other studies were not commonly recorded observations [15,17-22].

The mean packed cell volume (PCV) and red blood cell (RBC) count were lower than the reference values. Waner *et al*. [23] also found low PCV and RBC in experimentally *E. canis-*infected dogs, but with no consistency. Similarly, platelet counts were lower than the reference values. Platelet count has been shown to be a good screening test for *E. canis* infection, with increasing reliability in relation to the degree of thrombocytopenia [24]. It is also used to assess recovery from the disease [25]. Platelet counts may be lower than normal because of the presence of anti-platelet serum during CME infection [26]. Meanwhile, differential and absolute counts of the white blood cells were within normal range, except for the absolute count of basophils. Basophil counts were found significantly higher in dogs infected with the closely related *Anaplasma platys* [27]. Basophils appear to participate in parasitic infections [28], but further studies are needed to determine its role in CME infection.

The average neutrophil to lymphocyte (N/L) ratio was 5.0 (standard deviation [SD]=9.2). It was found to be a significant predictor on the outcome of the disease (p=0.011). Those which reportedly recovered or died from the disease had an average N/L value of 3.7 (SD=4.1) and 12.0 (SD=20.1), respectively. To the best of the author’s knowledge, this study is the first to describe N/L as a predictor of CME outcome in dogs. The N/L has been used as a good prognostic marker that may also express disease severity [29].

Creatinine and blood urea nitrogen (BUN) appears to be elevated (Table-2). These are similar observations by that of Mylonakis *et al*. [22]. Although the mean alanine aminotransferase (ALT) value in this study was in normal range, its variability is very high between patients. Elevated ALT is a known observation in CME cases [19,22], which may be caused by liver-associated problems caused by the infection [30].

**Table-2:** Hematological and biochemical values of *E. canis*-seropositive dogs in Metro Cebu, Philippines.

Parameter	Mean	SD	Reference values
Packed cell volume (%)	33.6	16.9	35-57
Hemoglobin (g/dL)	22.1	89.1	12-19
RBC (×10^6^/μL)^a^	4.7	1.8	5.0-7.9
Platelet (×10^3^/μL)	110.9	93.5	211-621
White blood cell (×10^3^/μL)	13.1	9.7	5.0-14.1
Differential count (%)			
Basophil	5.6	10.5	0-1
Eosinophil	7.2	13.8	0-9
Neutrophil	65.0	22.0	58-85
Monocyte	5.3	6.1	2-10
Lymphocyte	24.1	18.1	8-21
Absolute count (×10^3^/μL)			
Basophil	0.4	0.5	0-0.1
Eosinophil^b^	0.9	1.6	0-1.3
Neutrophil^a^	0.7	1.1	0.1-1.4
Monocyte	2.9	3.0	0.4-2.9
Lymphocyte^a^	9.0	8.4	2.9-12
Alanine aminotransferase (u/L)	70.4	102.8	10-109
Creatinine (mg/dL)	3.6	8.9	0.5-1.7
Blood urea nitrogen (mg/dL)	29.3	48.7	8.0-28.0

a Weak correlation with platelet count, p<0.05,

b Weak correlation with RBC count, p<0.05.

RBC=Red blood cell, SD=Standard deviation, *E. canis=Ehrlichia canis*

The most common clinical signs observed were inappetence (41.3%), lethargy/depression (35.6%), vomiting (32.4%), fever (18.5%), paleness (8.2%), and epistaxis (6.6%) (Table-3). In other studies, lethargy, epistaxis, apathy, anorexia, pale mucous membrane, lymphadenopathy, splenomegaly, and uveitis [30,31] were also observed. However, fever was not frequently observed by Inokuma *et al*. [20]. In a study by Stockham *et al*. [16], fever was shown to occur at 18-24 days after *E. canis* inoculation. In the same manner, epistaxis was not consistently seen in infected patients. David *et al*. [32] suggested that it is the most dramatic sign of the disease in experimentally infected German shepherd dogs. Based on records, it can be deduced that not all the observed clinical signs with the highest frequency are congruent with the other reports. Thus, clinical signs of CME appear to be varied and non-specific. This may be attributed to the infection of *E. canis* to circulating monocytes, which affects different body systems and produce varied clinical signs or combinations [6]. CME can be multi-systemic affecting several organs [32]. It may also be due to the different disease stages of the presented animals [6].

**Table-3:** Common clinical signs of *E. canis* seropositive dogs in Metro Cebu, Philippines (n=438).

Clinical sign	n	%
Inappetence		41.3
Lethargy/depression		35.6
Vomiting		32.4
Fever		18.5
Paleness*		8.2
Epistaxis*		6.6
Diarrhea		6.2
Coughing		5.9
Anorexia		3.2
Opthalmological lesions		3.0
Icterus		1.4
Neurological signs		0.9
Dyspnea		0.7
Edema of the limbs	3	0.7
Ocular discharges	3	0.7
Nasal discharges	2	0.5
Scrotal edema	2	0.5

* p<0.05;

Moderate correlation with RBC count of *E. canis* seropositive dogs. *E. canis=Ehrlichia canis*, RBC=Red blood cell

Further analyses showed that there were no significant differences on the other hematological parameters between thrombocytopenic and non-thrombocytopenic dogs. Moreover, very weak correlations were found between platelet count and RBC count (p=0.047, R=0.108, R^2^=0.012), absolute lymphocyte count (p=0.004, R=0.155, R^2^=0.024) and neutrophil count (p<0.000, R=0.210, R^2^=0.044). However, none of the clinical signs were found significantly related with the thrombocytopenic state of dogs. While PCV (p=0.000, R=0.787, R^2^=0.619) was found correlated with the RBC count, only paleness (p=0.008) and epistaxis (p=0.004) were found to be significantly different between anemic and non-anemic patients. In addition, eosinophil count (p=0.004, R=0.360, R^2^=0.130) was found to be weakly correlated with the RBC count. *Ehrlichial morulae* were already reported in the eosinophil of infected dogs [33]. Eosinophils are known to participate in the inflammation process [34] and parasitism [35].

There is a possibility that the clinical, hematological and biochemical findings of the patients were not only influenced by *E. canis*, but also by other pathogens transmitted by the same tick vector, including *A. platys* and *Babesia gibsoni*. Ticks have known the capability of hosting multiple pathogenic organisms [36], which can result in a co-infected dog [37]. Co-infection with other pathogens is not uncommon [22], which can produce severe signs [38] that can be fatal to the diseased patient [39]. It is difficult to associate a specific clinical sign or hematological abnormality to a particular canine vector-borne disease due to possibilities of co-infection [40]. Another consideration is the possibility of serological cross-reaction of *E. canis* with the zoonotic *Anaplasma phagocytophilum* [21]. With such assumption, some of the positive dogs may be infected with *A. phagocytophilum* instead of *E. canis*.

In all cases, peripheral blood smear examination (PBSE) was not recorded to be performed. Although with low reliability [41], PBSE as a method for detecting *Ehrlichia* infection is a relatively cheap alternative to the more expensive commercial test kits. Veterinarians should perform PBSE if condition warrants.

## Conclusion

*E. canis* infection in dogs may produce varied clinical signs that may be influenced by the thrombocytopenic and anemic states of affected animals. Complete blood counts remain important in the diagnosis of the disease, especially the platelet and RBC counts. Creatinine, BUN, and ALT can be of value in the diagnosis of the infection. Several cases were lost to follow-up and appeared to be a challenge for handling veterinarians to monitor compliance of owners and progress of infected patients.

## Authors’ Contributions

APY and RHDY conceptualized the study, and analyzed and wrote the manuscript. RRV contributed in the data analysis. HPFM, DNMB, SVN, and SMBO collected the information from the different clinics. All authors read and approved the final manuscript.
